# Hemp Seed Protein-Derived Lipase Inhibitory Peptides Attenuate High-Fat Diet-Induced Obesity: Evidence from Intestinal Fat Digestion and Gut–Liver Axis Regulation

**DOI:** 10.3390/foods15112040

**Published:** 2026-06-05

**Authors:** Hao Yin, Jiangxiong Zhu, Ruilong Luo, Yu Zhong, Ting Zhao, Minyan Zhang, Yun Deng

**Affiliations:** 1 Department of Food Science & Technology, Shanghai Jiao Tong University, 800 Dongchuan Road, Shanghai 200240, China; yinhao12138@sjtu.edu.cn (H.Y.); zjx261023@163.com (J.Z.); zhongyu@sjtu.edu.cn (Y.Z.); 2 Yunnan Dali Research Institute of Shanghai Jiao Tong University, Dali 671000, China; ybzt1993@163.com; 3 Eryuan County Inspection and Testing Institute, Dali 671299, China; 15025091524@163.com (R.L.); 15121112361@163.com (M.Z.)

**Keywords:** hemp seed protein, bioactive peptides, anti-obesity, pancreatic lipase inhibition, gut microbiota, gut-liver axis

## Abstract

Obesity and its metabolic complications represent a major global health challenge, and food-derived bioactive peptides are emerging as promising dietary interventions. In this study, two hemp seed protein-derived tetrapeptides with pancreatic lipase (PL) and cholesterol esterase (CE) inhibitory activity, APAM and RLPA, were co-administered with a high-fat diet (HFD) to male C57BL/6J mice at 25 and 100 mg/kg body weight for 10 weeks. Both peptides dose-dependently alleviated HFD-induced body weight gain, visceral fat accumulation, hepatic steatosis, dyslipidemia, hyperglycemia, and systemic inflammation. Mechanistically, both peptides inhibited intestinal PL and CE activities and enhanced fecal lipid excretion, supporting direct suppression of intestinal fat digestion. 16S rRNA gene sequencing revealed partial restoration of HFD-disrupted gut microbiota, with APAM preferentially enriching *Bifidobacterium* and *Roseburia*, while RLPA promoted *Akkermansia* and *Lactobacillus*, accompanied by differential improvements in fecal short-chain fatty acid (SCFA) profiles. Hepatic transcriptomics identified the PPAR signaling pathway as a shared regulatory hub, and multi-omics integration revealed significant correlations linking gut microbiota, SCFA production, hepatic gene expression, and metabolic phenotypes. These findings suggest a dual-pathway anti-obesity mechanism integrating intestinal lipid digestion inhibition with gut–liver axis modulation, and highlight hemp seed protein-derived peptides as potential functional food ingredients for obesity prevention.

## 1. Introduction

Obesity, which is mainly caused by chronic positive energy balance resulting from increased intake of high-caloric diets and reduced physical activity, has become one of the most pressing global public health challenges in recent decades [1]. The development of obesity is closely associated with the increased incidence of a series of metabolic diseases, including diabetes, cardiovascular disease, and non-alcoholic fatty liver disease, which collectively impose enormous health and economic burdens worldwide [2,3]. Although pharmacological interventions such as Orlistat and GLP-1 receptor agonists have shown clinical efficacy in obesity management, their long-term use is often accompanied by gastrointestinal side effects, cardiovascular risks, and reduced patient compliance, which greatly limit their widespread application [4]. Therefore, developing safe and effective food-derived bioactive components as dietary-based strategies to prevent and alleviate obesity-induced metabolic disorders is of great significance [5,6].

The digestion and absorption of dietary fat is a complex biochemical process that relies on the catalytic activity of multiple digestive enzymes. Among them, pancreatic lipase (PL), the key rate-limiting enzyme responsible for approximately 50–70% of dietary triglyceride hydrolysis, and cholesterol esterase (CE), which catalyzes the hydrolysis of cholesterol esters in the intestinal lumen, play indispensable roles in dietary fat and cholesterol absorption [7]. Inhibition of PL and CE activities can directly reduce dietary fat digestion and absorption, thereby lowering the lipid input to the body from the source, which represents a well-established anti-obesity strategy. Orlistat is the only FDA-approved lipase inhibitor for obesity management; however, its clinical utility is limited by gastrointestinal adverse effects, including steatorrhea, fecal urgency, and fat-soluble vitamin malabsorption [8]. Recently, food-derived bioactive peptides have attracted increasing attention as potential natural PL and CE inhibitors due to their natural origin, high safety profile, wide availability, and multi-target regulatory potential. Several studies have reported lipase inhibitory peptides derived from oyster protein [9], brewer’s spent grain protein [10], and other food protein sources. For instance, Ferreira et al. (2022) [10] demonstrated that microencapsulated brewer’s spent grain peptides could preserve their in vivo hypolipidemic activity by enhancing the inhibition of both PL and CE under simulated gastrointestinal digestion. However, most of the current studies have focused on in vitro enzyme inhibitory activity screening and characterization, and direct in vivo evidence for lipase inhibitory peptides in suppressing intestinal fat digestion remains scarce. In particular, whether oral administration of lipase inhibitory peptides can systematically ameliorate obesity-related metabolic disorders and through which underlying mechanisms this may be possible is still largely unknown.

Accumulating evidence reveals that gut microbiota plays a pivotal role in regulating host energy homeostasis and lipid metabolism, and the alteration of gut microbial composition has been closely associated with the development of obesity and related metabolic dysfunction [11]. High-fat diet feeding has been shown to significantly alter gut microbiota diversity, typically manifested as an elevated Firmicutes to Bacteroidota (F/B) ratio and decreased α-diversity, which are considered important triggering factors for HFD-induced metabolic disorders [12,13,14]. Meanwhile, short-chain fatty acids (SCFAs), the major metabolites produced by gut microbiota, can be transported to the liver via portal vein circulation, activating G protein-coupled receptors (GPR41/GPR43) and the PPAR signaling pathway to regulate hepatic de novo lipogenesis and fatty acid β-oxidation [15,16]. Recently, many food-derived bioactive peptides have been reported to modulate HFD-induced gut dysbiosis and improve obesity-related metabolic disorders. For instance, fish collagen peptides improved gut microbial composition in obese mice by reducing the F/B ratio [12], and walnut peptides alleviated HFD-induced obesity by remodeling gut microbiota and regulating SCFA contents [14]. Quinoa peptides and corn peptides were also found to attenuate HFD-induced obesity through regulating hepatic PPAR signaling and gut microbiota structure [2,17], and sturgeon-derived peptide LLLE was reported to reverse lipid accumulation by modulating gut microbiota balance and hepatic transcriptional pathways [18]. However, systematic multi-omics evidence integrating gut microbial composition changes, SCFA metabolic profiles, and hepatic transcriptome responses is still lacking to clarify whether food-derived peptides can exert anti-obesity effects through the complete “gut microbiota-SCFAs-hepatic PPAR pathway” gut-liver axis cascade.

In our previous work, two tetrapeptides with dual PL and CE inhibitory activities, APAM (Ala-Pro-Ala-Met) and RLPA (Arg-Leu-Pro-Ala), were identified and screened from hemp seed protein hydrolysates using a peptidomics and virtual screening combined strategy [19], and their molecular binding modes and interaction mechanisms with PL and CE were systematically elucidated using enzyme kinetics, fluorescence spectroscopy, isothermal titration calorimetry, and molecular docking [20]. It is worth noting that lipase inhibitory peptides may exert anti-obesity effects not only by directly inhibiting intestinal PL and CE activities to reduce dietary fat absorption, but also by reaching the colon to remodel gut microbiota composition and modulate SCFA production, which in turn improves hepatic lipid metabolism through the gut-liver axis. However, whether these two peptides could exert anti-obesity effects in vivo and modulate gut microbiota and hepatic lipid metabolism to alleviate HFD-induced metabolic disorders had not been reported yet. Moreover, no study to date has simultaneously investigated the anti-obesity mechanisms of lipase inhibitory peptides from both the “direct enzyme inhibition” and “gut-liver axis modulation” dimensions. Therefore, this study aimed to investigate the effects of APAM and RLPA on HFD-induced obesity and related metabolic disorders in C57BL/6J mice. Multiple analytical approaches, including intestinal digestive enzyme activity assay, fecal fat excretion measurement, 16S rRNA gene sequencing, fecal SCFAs targeted metabolomics, and hepatic transcriptomics (RNA-seq), were integrated to systematically elucidate the anti-obesity mechanisms of two hemp seed-derived lipase inhibitory peptides with different inhibitory potencies. This research would provide a novel insight into food-derived lipase inhibitory peptides in alleviating HFD-induced obesity-related metabolic disorders from the perspective of integrated multi-omics and thus establish a theoretical basis for developing hemp seed-derived bioactive peptides into functional food ingredients or dietary supplements for obesity prevention.

## 2. Materials and Methods

### 2.1. Materials and Reagents

Two peptides, APAM and RLPA, with a purity ≥95%, were synthesized by Nanjing PeptideValley Biotechnology Co., Ltd. (Nanjing, China) (Figure S1). The assay kits for serum total cholesterol (TC), triglyceride (TG), low-density lipoprotein cholesterol (LDL-C), high-density lipoprotein cholesterol (HDL-C), insulin, alanine aminotransferase (ALT) and aspartate aminotransferase (AST), as well as the ELISA kits for interleukin-6 (IL-6) and tumor necrosis factor-α (TNF-α) were all purchased from Nanjing Jiancheng Bioengineering Institute (Nanjing, China). Unless otherwise specified, all chemical reagents used in this study were of analytical grade.

### 2.2. Animal Experiment and Grouping Design

The animal experimental protocol was approved by the Experimental animal ethics committee of Shanghai Jiaotong University (Approval number: A2023184). Four-week-old SPF grade male C57BL/6J mice were purchased from Shanghai SLAC Laboratory Animal Co., Ltd. (Shanghai, China) and housed in a barrier-grade animal facility under controlled environmental conditions (temperature 24 ± 2 °C, relative humidity 50 ± 5%, 12 h light/12 h dark cycle) with ad libitum access to food and water throughout the experimental period. After one week of acclimation, mice were randomly allocated into six groups (*n* = 8 per group) based on body weight matching. Randomization was performed using a body weight-stratified method. After acclimation, all mice were ranked by body weight and sequentially assigned to six groups to ensure comparable baseline body weights across groups. Mice were housed at 4 animals per cage (2 cages per group) under standard conditions. Cage assignment within each group was random. The investigator performing daily gavage was aware of group assignments due to the nature of the intervention; however, outcome assessments, including body weight recording, sample collection, biochemical analyses, and histopathological evaluation, were conducted by researchers blinded to group allocation. No animals were excluded from the study; all 48 mice (*n* = 8 per group) survived to the endpoint without exhibiting signs of illness requiring humane endpoints, and all were included in the final analyses. The normal diet control group (ND group) was fed a chow diet (XTCON50J, 10% kcal from fat, Jiangsu Xietong Pharmaceutical Bioengineering Co., Ltd., Nanjing, China) throughout the entire experimental period and received daily oral gavage of an equivalent volume of PBS. The remaining five groups were all fed a high-fat diet (HFD; XTHF60, 60% kcal from fat, the same supplier; detailed diet compositions were provided in Table S1) to induce obesity, while simultaneously receiving the following gavage interventions: HFD model group (equivalent volume of PBS), low-dose APAM group (L-APAM, 25 mg/kg body weight), high-dose APAM group (H-APAM, 100 mg/kg body weight), low-dose RLPA group (L-RLPA, 25 mg/kg body weight) and high-dose RLPA group (H-RLPA, 100 mg/kg body weight). To ensure maximal peptide integrity during the intervention period, all peptide gavage solutions were freshly prepared on a daily basis. The lyophilized peptide powders were stored desiccated at −20 °C in sealed containers protected from light and moisture. Each morning, the required concentration for each treatment group was calculated based on the most recently recorded body weight of each individual mouse and the corresponding gavage volume (≤200 μL per mouse, adjusted according to individual body weight), and the peptides were dissolved in sterile PBS (pH 7.4) immediately before oral administration.

### 2.3. Experimental Procedure and Sample Collection

**Figure 1:**
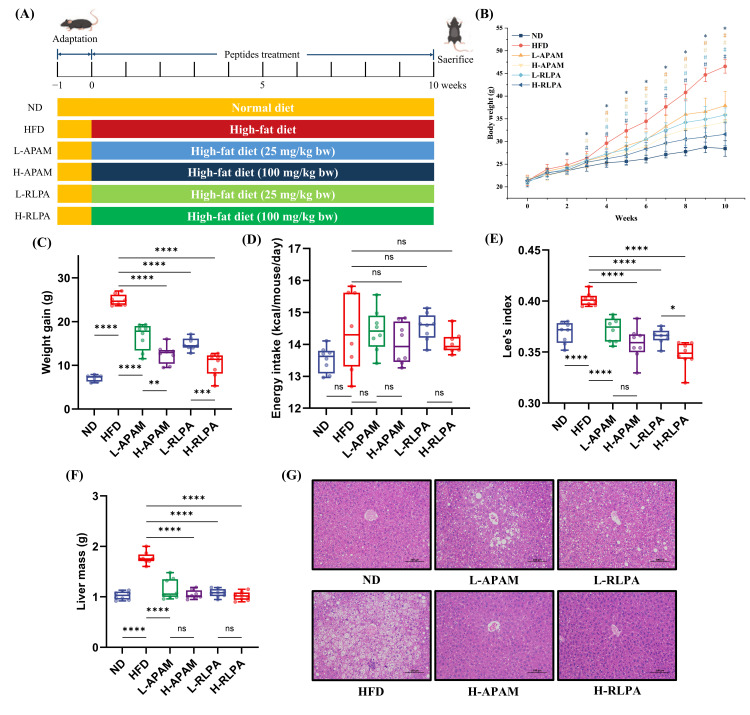
Effects of APAM and RLPA supplementation on HFD-induced obesity in mice. (**A**) Schematic diagram of the experimental design and grouping strategy. (**B**) Body weight changes over the 10-week intervention period. (**C**) Body weight gain. (**D**) Average daily energy intake. Food consumption was measured per cage and averaged per mouse (2 cages per group, 4 mice per cage; each data point represents one measurement interval of approximately two weeks, yielding 8 data points per group). (**E**) Lee’s index. (**F**) Liver mass. (**G**) Representative H&E-stained images of liver sections. Data are expressed as mean ± SD (*n* = 8). * *p* < 0.05, ** *p* < 0.01, *** *p* < 0.001, **** *p* < 0.0001; ns, not significant.

The complete experimental scheme and the timeline of peptide intervention were illustrated in Figure 1A. The intervention period lasted for 10 weeks, during which the body weight of each individual mouse was recorded weekly. Food consumption was measured on a per-cage basis (4 mice/cage, 2 cages per group) over approximately two-week intervals throughout the 10-week experimental period, and the average daily energy intake per mouse was calculated by dividing total cage food consumption by the number of mice and the number of days in each measurement interval, yielding 8 measurement points per group across the entire intervention period. Fresh fecal samples were collected once at the end of week 10 (the terminal time point only) and immediately stored at −80 °C until further analysis. At the end of the experiment, mice were fasted for 12 h, and fasting blood glucose levels were determined from tail vein blood. The naso-anal body length was also measured and Lee’s index was calculated accordingly [21]. Subsequently, the mice were anesthetized by isoflurane inhalation and euthanized. Whole blood, liver, intestinal tissues and multiple adipose depots were rapidly dissected and collected, including epididymal white adipose tissue (epiWAT), inguinal white adipose tissue (ingWAT) and mesenteric white adipose tissue (mesWAT). All tissues were accurately weighed and then processed: one portion was snap-frozen in liquid nitrogen and transferred to a −80 °C ultra-low temperature freezer for subsequent analysis, while another portion was immersed in 4% paraformaldehyde fixative for histological examination.

### 2.4. Determination of Serum Biochemical Indexes

Whole blood was collected into non-anticoagulant vacuum blood collection tubes and centrifuged at 3000× *g* for 10 min at 4 °C to separate serum, which was then aliquoted and stored at −80 °C until analysis. The serum levels of TC, TG, HDL-C, LDL-C, insulin, ALT and AST were quantitatively determined using the corresponding commercial assay kits from Nanjing Jiancheng Bioengineering Institute according to the manufacturer’s instructions. The serum concentrations of IL-6 and TNF-α were detected by ELISA following the procedures provided with the respective kits.

### 2.5. Determination of Hepatic Lipid Contents

Liver tissue (0.1 g) was precisely weighed and homogenized in 0.9 mL anhydrous ethanol. The homogenate was centrifuged at 10,000× *g* for 10 min, and the supernatant was collected for the determination of TC and TG contents using the same assay kits as described in Section 2.4.

### 2.6. Determination of Fecal Lipid Contents

Fecal samples (0.2 g) were weighed and mixed with 1 mL of 60% ethanol extraction solution containing 0.2% glacial acetic acid. The mixture was ground, homogenized and vortexed for 5 min, followed by ultrasonication for 30 min to ensure thorough lipid extraction. After centrifugation at 10,000× *g* for 10 min, the supernatant was collected, and the TC and TG contents were determined using the assay kits from Nanjing Jiancheng Bioengineering Institute.

### 2.7. Determination of Total Intestinal Lipid Digestive Enzyme Activity

The activities of lipid digestion-related enzymes in the duodenal contents were measured according to the method reported by Ferreira et al. (2022) [10]. The total protein concentration of each sample was determined by the BCA method, and the enzyme activities were normalized and expressed as U/g protein. It should be noted that the p-nitrophenyl butyrate substrate used in this assay can be hydrolyzed by both PL and CE; therefore, the measured activity represents the total lipid hydrolytic capacity of the intestinal contents rather than the activity of a single enzyme.

### 2.8. Histopathological Analysis

The liver and adipose tissue samples fixed in 4% paraformaldehyde were subjected to routine dehydration, clearing, and paraffin embedding. Serial paraffin sections of 4 μm thickness were prepared using a microtome (Thermo Fisher Scientific, Waltham, MA, USA) and stained following the standard hematoxylin and eosin (H&E) staining protocol. The stained sections were observed and images were captured using an Olympus IX50 inverted optical microscope (Tokyo, Japan).

### 2.9. 16S rRNA Gene Sequencing of Gut Microbiota

The microbial community structure of fecal samples (approximately 100 mg per sample) was analyzed by Shanghai Personalbio Technology Co., Ltd. (Shanghai, China). Total genomic DNA was extracted using the OMEGA Soil DNA Kit (M5635-02, OMEGA Bio-Tek, Norcross, GA, USA). The V3–V4 hypervariable region of the bacterial 16S rRNA gene was amplified by PCR using the forward primer 338F (5′-ACTCCTACGGGAGGCAGCA-3′) and the reverse primer 806R (5′-GGACTACHVGGGTWTCTAAT-3′). The amplification products were quantified and pooled in equimolar ratios, and then subjected to paired-end sequencing (2 × 250 bp) on the Illumina NovaSeq platform (Illumina, San Diego, CA, USA) using the NovaSeq 6000 SP Reagent Kit (500 cycles, Illumina, San Diego, CA, USA). Quality control and bioinformatic processing of the raw sequencing data were performed based on the QIIME2 pipeline (version 2022.11), and the data visualization was accomplished using the Personalbio GenesCloud online platform (https://www.genescloud.cn (accessed on 15 March 2026)).

### 2.10. Determination of Fecal Short-Chain Fatty Acids (SCFAs)

The concentrations of SCFAs in fecal samples were quantified using an internal standard method with slight modifications as previously described by Zhu et al. (2026) [22]. Briefly, 50 mg of fecal sample was precisely weighed into a centrifuge tube and homogenized with 200 μL ultrapure water for 60 s. After centrifugation at 4000× *g* for 10 min, the supernatant was collected. An aliquot of 0.8 mL supernatant was mixed sequentially with 0.1 mL of 50% sulfuric acid solution and 1 mL of internal standard working solution (50 μg/mL), and the mixture was allowed to stand at 4 °C for 30 min to achieve liquid–liquid extraction and phase separation. The upper organic phase was transferred into a sample vial and analyzed using an Agilent 6890 gas chromatograph (Agilent Technologies, Santa Clara, CA, USA) equipped with a flame ionization detector (FID). The chromatographic conditions were as follows: a DB-FFAP capillary column; injection volume of 1 μL; injector temperature of 220 °C; initial column temperature of 100 °C; detector temperature of 250 °C; carrier gas of high-purity nitrogen (purity ≥ 99.999%) at a flow rate of 2 mL/min. All SCFA standards (purity ≥ 98%) were purchased from Shanghai Saan Chemical Technology Co., Ltd. (Shanghai, China).

### 2.11. Hepatic Transcriptome Sequencing

The transcriptome sequencing of liver tissue was performed by Shanghai Personalbio Technology Co., Ltd. (Shanghai, China) [23]. Total RNA was extracted from liver tissue using TRIzol reagent (Invitrogen, Carlsbad, CA, USA). Briefly, the tissue was thoroughly homogenized and subjected to chloroform-based phase separation. After centrifugation, the aqueous phase was collected and RNA was precipitated with an equal volume of isopropanol. The RNA pellet was washed with 75% ethanol and dissolved in RNase-free water. The concentration and purity of total RNA were assessed using a NanoDrop 2000 spectrophotometer (Thermo Fisher Scientific, Waltham, MA, USA), and the RNA integrity was evaluated by agarose gel electrophoresis or an Agilent 2100 Bioanalyzer (Agilent Technologies, Santa Clara, CA, USA) coupled with an RNA 6000 Nano Kit (Agilent Technologies, Santa Clara, CA, USA).

The sequencing library was constructed from no less than 1 μg of qualified total RNA using the NEBNext Ultra II RNA Library Prep Kit for Illumina (New England Biolabs, Ipswich, MA, USA). Poly(A)+ mRNA was first enriched using Oligo(dT) magnetic beads, followed by fragmentation, double-stranded cDNA synthesis, end repair, 3′ adenylation and sequencing adaptor ligation. AMPure XP magnetic beads were used to select insert fragments of 400–500 bp, and the final library was obtained after PCR amplification and purification. The library quality was assessed by an Agilent 2100 Bioanalyzer (High Sensitivity DNA Kit) (Agilent Technologies, Santa Clara, CA, USA) to determine the fragment size distribution. The total library concentration was quantified by PicoGreen fluorescence assay, and the effective concentration was further validated by qPCR. The validated libraries were pooled in equimolar ratios, diluted to the appropriate concentration, and sequenced on the Illumina platform in the PE150 mode.

The raw sequencing data were subjected to standard quality control and alignment procedures. Differentially expressed genes (DEGs) were identified using the criteria of |log_2_FoldChange| > 1 and *p* < 0.05. Kyoto Encyclopedia of Genes and Genomes (KEGG) pathway enrichment analysis was performed using the R package ClusterProfiler (v4.6.0) with a significance threshold of *p* < 0.05. The data visualization was accomplished using the Personalbio GenesCloud online platform (https://www.genescloud.cn (accessed on 20 March 2026)).

### 2.12. qRT-PCR Validation

To verify the reliability of the transcriptome sequencing results, six representative DEGs, including Cyp2b9 and Cyp2b13, were selected for qRT-PCR analysis [24]. The primer sequences were designed using the Primer3 online tool and were listed in Supplementary Table S2. Total RNA was extracted from the liver tissue of each group using TRIzol reagent (three mice were randomly selected per group, *n* = 3), and reverse-transcribed into cDNA using SuperScript II reverse transcriptase. With β-actin serving as the internal reference gene, qPCR amplification was carried out using PowerUp™ SYBR Green Master Mix on an ABI Prism 7500 Real-Time PCR system (Applied Biosystems, USA). The reaction was performed in a total volume of 20 μL under the following thermal cycling conditions: initial denaturation at 95 °C for 10 s, followed by 40 cycles of 95 °C for 10 s and 58 °C for 30 s (annealing/extension). Each sample was analyzed in triplicate, and a no-template negative control was included. The specificity of each primer pair was confirmed by melt curve analysis, which showed a single sharp peak for all amplicons without primer dimers or non-specific products. All amplification curves displayed typical sigmoid profiles with consistent baseline and exponential phases, and no-template controls showed no amplification throughout 40 cycles, confirming the absence of contamination. β-Actin was selected as the internal reference gene based on its widely documented expression stability in mouse liver tissue under HFD conditions [9,24], and its stable expression across all experimental groups in the present study was verified by consistent Ct values. The relative expression levels of target genes were calculated using the 2^−ΔΔCT^ method.

### 2.13. Multi-Omics Integrative Analysis

To systematically evaluate the intrinsic associations among gut microbiota, fecal short-chain fatty acids, hepatic transcriptome and host metabolic phenotypes, a multi-omics integrative analysis was performed using a combined strategy of Mantel test and Spearman correlation analysis [25].

Mantel tests were employed to evaluate the overall associations between different omics datasets and the metabolic phenotype matrix. Bray–Curtis distance matrices were constructed from the relative abundance of gut microbiota at the genus level, while Euclidean distance matrices were generated from SCFA concentrations (total SCFAs and individual SCFAs), hepatic gene expression profiles, and selected gene expression levels (e.g., Pparg), respectively. These distance matrices were then compared with the Euclidean distance matrix derived from 16 metabolic phenotypic parameters (including body weight gain, adipose tissue weight, serum lipid profiles, blood glucose, liver function indices and inflammatory cytokines) via Mantel tests with 9999 permutations. Pearson correlation coefficients were simultaneously calculated among the metabolic indicators to visualize their intercorrelations. The significance threshold was defined as *p* < 0.05.

Spearman rank correlation analyses were further performed to assess the pairwise associations across different omics layers, including: (i) significantly altered genera identified from 16S rRNA gene sequencing versus metabolic phenotypic parameters; (ii) differential genera versus six major fecal SCFAs (acetic acid, propionic acid, butyric acid, isobutyric acid, valeric acid and isovaleric acid); and (iii) fecal SCFAs versus representative differentially expressed genes from key KEGG pathways in the hepatic transcriptome. The above analyses were performed using the Omicstudio (https://www.omicstudio.cn/tool) [26].

### 2.14. Statistical Analysis

The sample sizes for different analyses were as follows: *n* = 8 per group for body weight, organ weight, serum biochemistry, fecal lipid, and intestinal enzyme activity measurements; *n* = 6 per group for 16S rRNA gene amplicon sequencing and fecal SCFA quantification (except for the L-APAM group where *n* = 5 due to one sample failing quality control); *n* = 6 for the ND and HFD groups and *n* = 4 for the four peptide-supplemented groups for hepatic transcriptome sequencing (RNA-seq); and *n* = 3 per group for qRT-PCR validation. Data processing and statistical analysis were carried out using SPSS 27.0 (IBM, USA) and GraphPad Prism 9.0 (GraphPad Software, USA). Multiple group comparisons were performed by one-way analysis of variance (ANOVA), followed by Šidák’s multiple comparisons test for pairwise comparisons. A value of *p* < 0.05 was considered statistically significant. All experimental data were expressed as mean ± standard deviation (SD).

## 3. Results

### 3.1. APAM and RLPA Supplementation Effectively Prevented HFD-Induced Obesity in Mice

To evaluate the in vivo anti-obesity effects of the two lipase inhibitory peptides APAM and RLPA, C57BL/6J mice were fed a high-fat diet (HFD) and simultaneously administered each peptide at low (25 mg/kg bw) or high (100 mg/kg bw) doses by daily gavage for 10 weeks (Figure 1A). As shown in Figure 1B, no significant differences in initial body weight were observed among the six groups. The body weight of mice in the HFD group increased rapidly and became significantly higher than that of the ND group from week 2 onward (*p* < 0.05). In contrast, the body weight curves of all four peptide-supplemented groups fell between those of the ND and HFD groups and progressively diverged from the HFD group starting from week 3, with the H-RLPA group displaying the slowest weight gain. Correspondingly, the cumulative body weight gain in the HFD group was approximately 3-fold that of the ND group (*p* < 0.0001, Figure 1C), confirming the successful establishment of the obesity model. All four peptide-supplemented groups showed significantly reduced body weight gain compared with the HFD group (all *p* < 0.0001), with reductions ranging from approximately 30% to 60%. Both peptides exhibited dose-dependent effects, and the RLPA groups consistently outperformed the corresponding APAM groups at each dose level. Notably, daily energy intake did not differ significantly among the six groups (Figure 1D). The Lee’s index was significantly elevated in the HFD group relative to the ND group (*p* < 0.0001, Figure 1E), and all peptide-supplemented groups showed significantly lower values (all *p* < 0.0001), with the H-RLPA group exhibiting the lowest value, even below that of the ND group.

HFD markedly increased the epididymal fat pad weight to approximately 4-fold that of the ND group (*p* < 0.0001, Figure S2A). All peptide-supplemented groups showed significantly reduced epididymal fat mass (all *p* < 0.0001), with the H-RLPA and H-APAM groups displaying the greatest reductions. H&E staining of epididymal adipose tissue confirmed that HFD induced pronounced adipocyte hypertrophy, which was attenuated to varying degrees in all peptide-supplemented groups, with the H-RLPA group showing morphology most comparable to the ND group (Figure S2B). Additionally, liver weight was significantly elevated in the HFD group (*p* < 0.0001, Figure 1F), and peptide supplementation reduced liver weight to levels comparable to the ND group (all *p* < 0.0001). Histological examination of liver sections revealed extensive lipid droplet vacuoles and disordered hepatocyte arrangement in the HFD group, whereas all peptide-supplemented groups, particularly the H-APAM and H-RLPA groups, showed markedly attenuated hepatic steatosis (Figure 1G). Taken together, both APAM and RLPA effectively prevented HFD-induced weight gain, visceral fat accumulation, liver enlargement and hepatic steatosis in a dose-dependent manner, with the H-RLPA group exhibiting the most pronounced anti-obesity effects.

### 3.2. APAM and RLPA Ameliorated HFD-Induced Dyslipidemia, Hepatic Lipid Accumulation, Liver Injury, Glucose Metabolic Disorders and Systemic Inflammation

**Figure 2:**
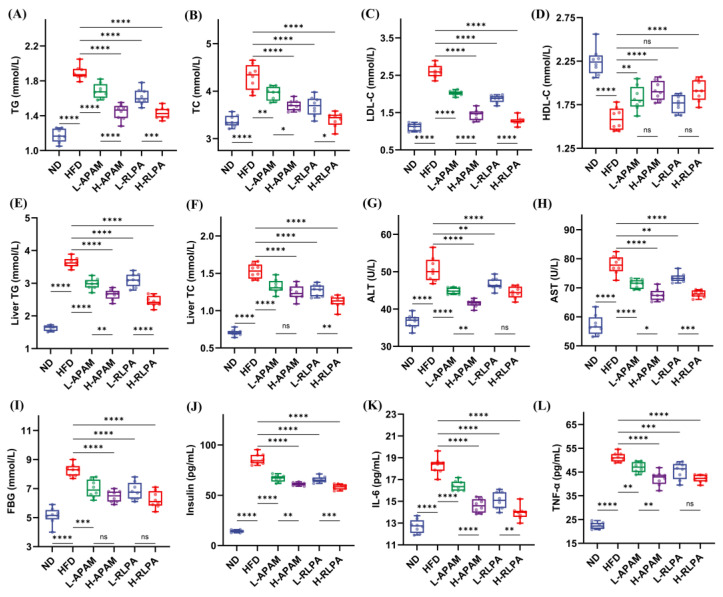
Effects of APAM and RLPA on serum and hepatic biochemical parameters. (**A**) Serum triglyceride (TG). (**B**) Serum total cholesterol (TC). (**C**) Serum low-density lipoprotein cholesterol (LDL-C). (**D**) Serum high-density lipoprotein cholesterol (HDL-C). (**E**) Hepatic TG content, expressed as concentration in the tissue homogenate supernatant. (**F**) Hepatic TC content, expressed as concentration in the tissue homogenate supernatant. (**G**) Serum alanine aminotransferase (ALT) activity. (**H**) Serum aspartate aminotransferase (AST) activity. (**I**) Fasting blood glucose (FBG). (**J**) Serum insulin level. (**K**) Serum interleukin-6 (IL-6) level. (**L**) Serum tumor necrosis factor-α (TNF-α) level. Data are expressed as mean ± SD (*n* = 8). * *p* < 0.05, ** *p* < 0.01, *** *p* < 0.001, **** *p* < 0.0001; ns, not significant.

To comprehensively assess the metabolic protective effects of the two peptides, serum lipid profiles, hepatic lipid contents, liver function markers, glucose metabolic parameters and serum inflammatory cytokines were determined (Figure 2).

Compared with the ND group, HFD significantly elevated serum TG, TC and LDL-C while reducing HDL-C (all *p* < 0.0001, Figure 2A–D). All four peptide-supplemented groups showed significantly lower serum TG levels than the HFD group (all *p* < 0.0001, Figure 2A). For TC and LDL-C, the high-dose groups exhibited more pronounced improvements, with the H-RLPA group showing TC levels essentially restored to those of the ND group and the greatest reduction in LDL-C among all groups (Figure 2B,C). HDL-C was significantly restored in the H-APAM and H-RLPA groups (*p* < 0.01 and *p* < 0.0001, respectively), whereas the two low-dose groups did not differ significantly from the HFD group (Figure 2D). Both peptides demonstrated dose-dependent improvements across all serum lipid parameters, and the RLPA groups generally showed superior efficacy to the corresponding APAM groups for TC and LDL-C.

HFD also induced significant hepatic lipid accumulation, with liver TG and TC contents markedly elevated relative to the ND group (both *p* < 0.0001, Figure 2E,F). All peptide-supplemented groups showed significantly reduced hepatic TG (all *p* < 0.01), with the H-RLPA group exhibiting the greatest reduction. Hepatic TC displayed a similar pattern. These biochemical findings were consistent with the attenuated hepatic steatosis observed in liver histology (Figure 1G).

Serum ALT and AST, classical indicators of hepatocellular damage, were significantly elevated in the HFD group (both *p* < 0.0001, Figure 2G,H). Peptide supplementation reduced both markers to varying degrees, with the H-APAM group showing the most significant decrease in ALT (*p* < 0.0001) and both H-APAM and H-RLPA groups significantly lowering AST (both *p* < 0.001).

HFD significantly elevated fasting blood glucose and serum insulin levels (both *p* < 0.0001, Figure 2I,J). All peptide-supplemented groups showed significantly lower values for both parameters compared with the HFD group (all *p* < 0.0001), although insulin levels remained substantially above those of the ND group. The H-RLPA group displayed the greatest reductions in both fasting glucose and insulin.

Furthermore, HFD markedly increased serum IL-6 and TNF-α levels (both *p* < 0.0001, Figure 2K,L). IL-6 was significantly reduced in all peptide-supplemented groups (all *p* < 0.0001), with the H-RLPA group approaching ND levels. The H-APAM and H-RLPA groups showed the most significant TNF-α reductions, though levels remained above those of the ND group. Overall, both peptides exhibited dose-dependent metabolic improvements, with the RLPA groups consistently showing greater efficacy than the corresponding APAM groups and the H-RLPA group displaying the most comprehensive ameliorative effects.

### 3.3. APAM and RLPA Inhibited Intestinal Fat Digestion and Promoted Fecal Fat Excretion In Vivo

**Figure 3:**
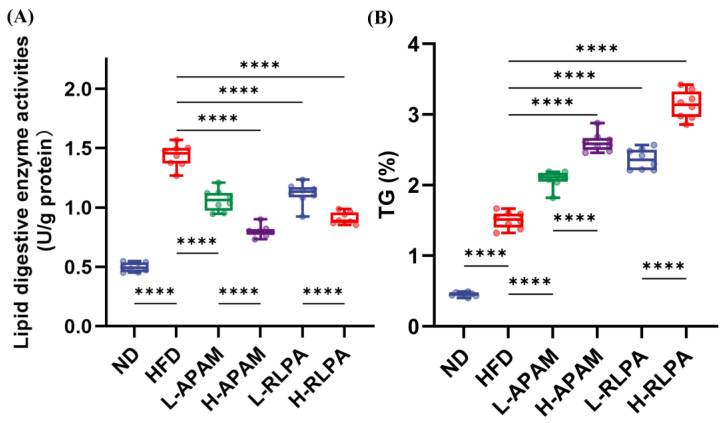
Effects of APAM and RLPA on total intestinal lipid digestive enzyme activity and fecal lipid excretion in HFD-fed mice. (**A**) Total intestinal lipid digestive enzyme activity. (**B**) Fecal triglyceride (TG) content. Data are expressed as mean ± SD (*n* = 8). **** *p* < 0.0001.

To determine whether the two lipase inhibitory peptides suppressed intestinal fat digestion in vivo, the total lipid digestive enzyme activity in small intestinal contents, which reflects the aggregate hydrolytic contributions of PL, CE, and other lipid-hydrolyzing enzymes and fecal triglyceride (TG) content were measured (Figure 3).

The total lipid digestive enzyme activity in the HFD group was approximately 1.9-fold that of the ND group (*p* < 0.0001, Figure 3A). All four peptide-supplemented groups exhibited significantly lower intestinal lipase activity than the HFD group (all *p* < 0.0001), directly demonstrating in vivo lipase inhibition by both peptides. Both peptides showed dose-dependent inhibitory effects, and notably, the APAM groups demonstrated stronger direct enzyme inhibition than the corresponding RLPA groups at each dose level.

Consistent with the reduced lipase activity, fecal TG content was significantly increased in all peptide-supplemented groups relative to the HFD group (all *p* < 0.0001, Figure 3B), indicating that peptide intervention effectively reduced intestinal fat digestion and absorption, resulting in increased fecal fat excretion. Both peptides displayed dose-dependent effects. Interestingly, although APAM exhibited stronger direct lipase inhibition, the RLPA groups showed significantly higher fecal fat excretion than the corresponding APAM groups at each dose level, revealing an apparent dissociation between the degree of enzyme inhibition and the extent of fecal fat output. These results demonstrated that both peptides inhibited intestinal fat digestion in vivo, and the discrepancy between enzyme inhibition potency and fecal fat output suggested that RLPA may suppress fat absorption through additional mechanisms beyond direct lipase inhibition.

### 3.4. APAM and RLPA Reshaped HFD-Disrupted Gut Microbiota and Modulated Fecal Short-Chain Fatty Acid Profiles

**Figure 4:**
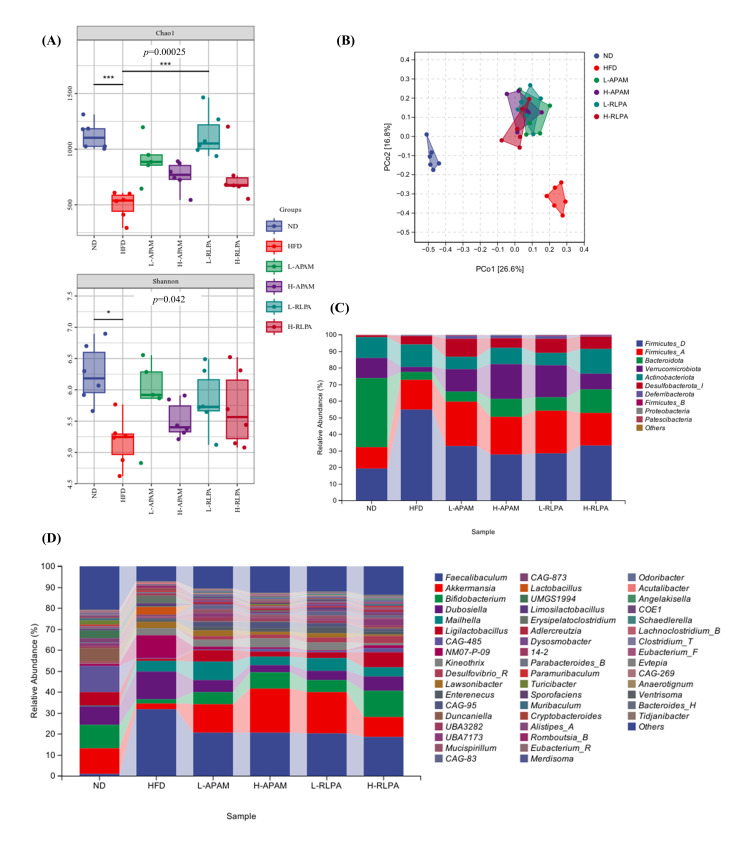
Effects of APAM and RLPA on gut microbiota diversity and composition in HFD-fed mice. (**A**) Alpha diversity indices (Chao1 and Shannon). (**B**) Principal coordinates analysis (PCoA) based on Bray–Curtis dissimilarity at the genus level. (**C**) Relative abundance of gut microbiota at the phylum level. (**D**) Relative abundance of gut microbiota at the genus level. Data are expressed as mean ± SD (*n* = 6). * *p* < 0.05, *** *p* < 0.001.

**Figure 5:**
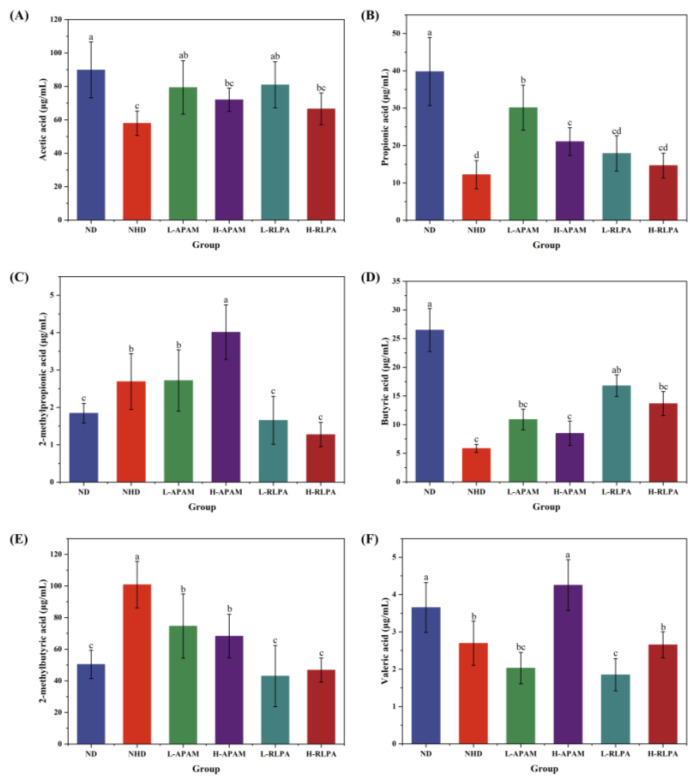
Effects of APAM and RLPA on fecal short-chain fatty acid (SCFA) profiles in HFD-fed mice. (**A**) Acetic acid. (**B**) Propionic acid. (**C**) Isobutyric acid. (**D**) Butyric acid. (**E**) Isovaleric acid. (**F**) Valeric acid. Data are expressed as mean ± SD (*n* = 6). Different lowercase letters indicate statistically significant differences among groups (*p* < 0.05).

To investigate the effects of APAM and RLPA on HFD-induced gut microbiota dysbiosis and its metabolic consequences, fecal 16S rRNA gene amplicon sequencing and quantification of fecal short-chain fatty acids (SCFAs) were performed (Figure 4, Figure 5, Figures S3 and S4).

The α-diversity of the gut microbiota was assessed using multiple indices (Figure 4A and Figure S3A). The Chao1 index showed highly significant differences among groups (*p* = 0.00025), with the HFD group exhibiting markedly reduced microbial richness compared with the ND group. All peptide-supplemented groups displayed higher Chao1 values than the HFD group, and the high-dose groups showed the most significant recovery (*p* < 0.001 vs. HFD), indicating a dose-dependent restoration effect. The Shannon index showed a consistent trend (*p* = 0.042), and the Observed species index further confirmed improved microbial richness (*p* = 0.00025, Figure S3A). The Simpson index showed an improving trend but did not reach statistical significance (*p* = 0.14). PCoA analysis based on Bray–Curtis distances (Figure 4B) revealed distinct separation between the ND and HFD groups, with all four peptide-supplemented groups clustering away from HFD and shifting toward ND. This pattern was validated by NMDS analysis (Figure S3B). Hierarchical clustering analysis (Figure S3D) confirmed that samples within each group clustered tightly, with the peptide-supplemented groups forming modules distinct from the HFD group. Venn diagram analysis (Figure S3C) identified 134 shared microbial features across all six groups, along with 3730, 1194, 1717, 1469, 2863 and 1580 group-specific features for the ND, HFD, L-APAM, H-APAM, L-RLPA and H-RLPA groups, respectively.

At the phylum level (Figure 4C), HFD induced a typical obesity-associated dysbiotic signature characterized by increased Firmicutes_D, decreased Bacteroidota and consequently an elevated F/B ratio, accompanied by an increasing trend of Desulfobacterota_I. Peptide supplementation partially reversed these alterations, restoring Bacteroidota abundance, reducing Firmicutes_D, and normalizing the F/B ratio. Notably, Verrucomicrobiota abundance was markedly increased in the peptide-supplemented groups, while Desulfobacterota_I was reduced. At the genus level (Figure 4D), several beneficial genera, including Akkermansia (particularly enriched in the RLPA groups), Bifidobacterium and Roseburia, showed increased abundance in the peptide-supplemented groups relative to the HFD group, whereas potentially harmful genera such as Desulfovibrio_R and Erysipelatoclostridium were reduced. Faecalibaculum, which was over-represented in the HFD group, declined after peptide supplementation, consistent with improved community evenness. Species-level analysis (Figure S4B) corroborated these observations.

LEfSe analysis (LDA threshold = 2.63, Figure S4C) identified distinct biomarkers for each group. The HFD group was characterized by taxa associated with sulfate reduction and obesity, including p_Firmicutes_D, g_Desulfovibrio_R, g_Faecalibaculum and g_Erysipelatoclostridium, while the ND group featured taxa indicative of a healthy gut ecosystem, such as p_Bacteroidota, g_Muribaculum, g_Duncaniella and g_Alistipes_A. The two peptides enriched distinct sets of beneficial genera: APAM groups preferentially enriched Bifidobacterium and Roseburia, whereas RLPA groups were characterized by enrichment of Lactobacillus, Ligilactobacillus and Akkermansia. KEGG functional pathway prediction (Figure S4D) indicated that peptide supplementation altered the functional potential of the gut microbiota, with differential trends observed in carbohydrate metabolism, lipid metabolism and cofactor and vitamin metabolism pathways relative to the HFD group.

The concentrations of six SCFAs in fecal contents were determined (Figure 5). HFD significantly reduced acetic acid, propionic acid and butyric acid levels compared with the ND group. For acetic acid, the L-APAM and L-RLPA groups showed the most effective restoration to levels comparable to the ND group. For propionic acid, the APAM groups demonstrated notably superior recovery over the RLPA groups. Conversely, the RLPA groups were more effective in restoring butyric acid, with the L-RLPA group reaching approximately 64% of the ND level. Unlike straight-chain SCFAs, branched-chain SCFAs, including isobutyric acid and 2-methylbutyric acid were elevated in the HFD group. The RLPA groups effectively normalized both branched-chain SCFAs to ND levels, whereas the APAM groups failed to reduce these levels and even further increased isobutyric acid. For valeric acid, only the H-APAM group showed significant recovery. Collectively, both peptides modulated fecal SCFAs profiles with characteristic differences: APAM preferentially restored propionic acid, while RLPA excelled in butyric acid recovery and branched-chain SCFAs normalization.

### 3.5. Hepatic Transcriptome Analysis Revealed That Both Peptides Commonly Regulated Lipid Metabolism and Inflammation-Related Signaling Pathways

**Figure 6:**
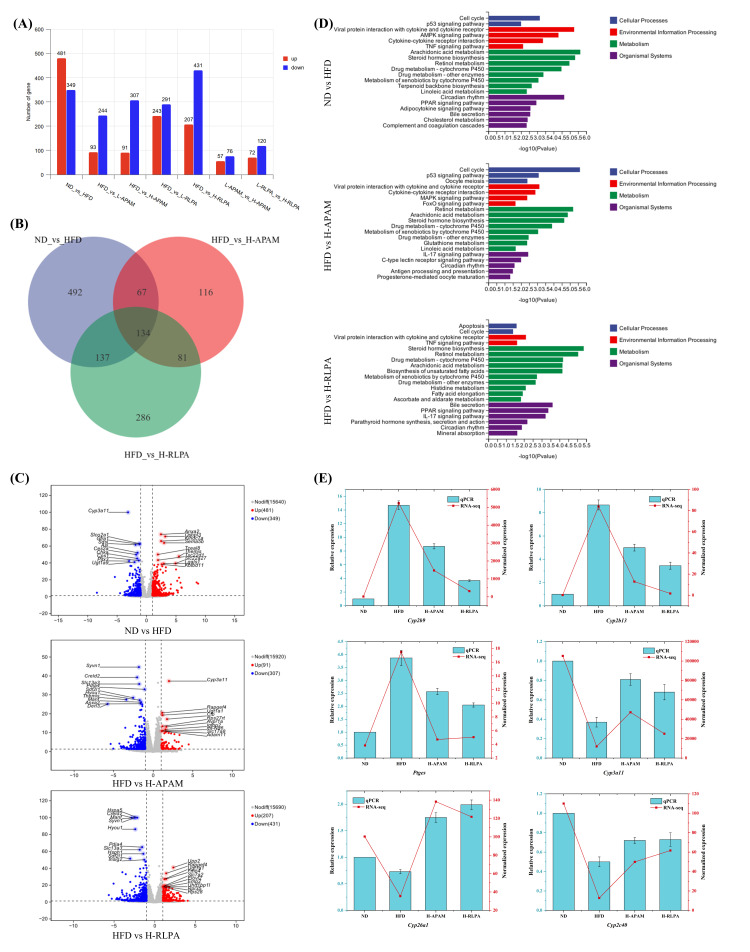
Hepatic transcriptome profiling in HFD-fed mice with APAM and RLPA intervention. (**A**) Number of up-regulated and down-regulated differentially expressed genes (DEGs) in each pairwise comparison. (**B**) Venn diagram showing overlapping DEGs among HFD vs. H-APAM and HFD vs. H-RLPA comparisons relative to the ND vs. HFD comparison. (**C**) Volcano plots of DEGs in ND vs. HFD (top), HFD vs. H-APAM (middle) and HFD vs. H-RLPA (bottom) comparisons, the horizontal dashed lines indicate the significance threshold, and the vertical dashed lines indicate the log_2_(Fold Change) thresholds. (**D**) KEGG pathway enrichment analysis of DEGs in ND vs. HFD (top), HFD vs. H-APAM (middle) and HFD vs. H-RLPA (bottom) comparisons. (**E**) Quantitative real-time PCR (qRT-PCR) validation of six representative DEGs. Data are expressed as mean ± SD (*n* = 3).

To characterize the transcriptomic effects of APAM and RLPA on the liver, hepatic tissues from all six groups were subjected to RNA-seq analysis (Figure 6 and Figure S5).

PCA of the whole transcriptome (Figure S5A) showed clear separation between the ND and HFD groups, with PC1 and PC2 explaining 32.8% and 19.7% of the total variance, respectively. The peptide-supplemented groups were positioned between the ND and HFD clusters with a shift toward ND, and the high-dose groups were located farther from HFD than their respective low-dose counterparts. The two low-dose groups and the two high-dose groups showed considerable spatial overlap, suggesting convergent transcriptomic effects between the two peptides. Under stringent criteria, 830 DEGs (481 up-regulated and 349 down-regulated) were identified in the ND vs. HFD comparison (Figure 6A,C). The H-RLPA vs. HFD and H-APAM vs. HFD comparisons yielded 638 DEGs (207 up, 431 down) and 398 DEGs (91 up, 307 down), respectively. Both high-dose groups exhibited a predominantly down-regulatory pattern (77.1% for H-APAM and 67.6% for H-RLPA). Across all three comparisons, Cyp3a11 consistently emerged as one of the most prominently altered genes. Hierarchical clustering of the full transcriptome organized genes into nine co-expression modules, with the peptide-supplemented groups displaying intermediate expression profiles between the ND and HFD groups (Figure S5B).

Venn analysis (Figure 6B) identified 134 core DEGs shared among the ND vs. HFD, HFD vs. H-APAM and HFD vs. H-RLPA comparisons, representing genes simultaneously altered by HFD and reversed by both peptides. Among the 830 HFD-responsive DEGs, 24.2% were reversed by H-APAM and 32.7% by H-RLPA. An additional 81 DEGs were shared exclusively between the two peptide comparisons but absent from the ND vs. HFD comparison. H-APAM and H-RLPA also possessed 116 and 286 unique DEGs, respectively.

GO enrichment analysis (Figure S5C) revealed highly consistent functional themes across all three comparisons. Biological process categories were dominated by lipid metabolism-related terms including unsaturated fatty acid metabolic process, fatty acid metabolic process, icosanoid metabolic process and arachidonic acid metabolic process. Molecular function categories consistently highlighted monooxygenase activity and oxidoreductase activity. Beyond the shared themes, H-APAM showed strong enrichment for inflammatory response processes, while H-RLPA displayed broader coverage of organic acid metabolic processes and notable enrichment for endoplasmic reticulum in the cellular component category.

KEGG pathway analysis (Figure 6D) identified arachidonic acid metabolism, steroid hormone biosynthesis, retinol metabolism and cytochrome P450-mediated pathways as commonly enriched across all three comparisons. The PPAR signaling pathway was significantly enriched in both the ND vs. HFD and HFD vs. H-RLPA comparisons, while the functionally related FoxO and MAPK signaling pathways were uniquely enriched in the HFD vs. H-APAM comparison. H-RLPA additionally showed unique enrichment for biosynthesis of unsaturated fatty acids and fatty acid elongation pathways. Both peptides regulated inflammation-related signaling pathways through different routes: C-type lectin receptor signaling for H-APAM and TNF and IL-17 signaling for H-RLPA. Bile secretion was enriched in both the ND vs. HFD and HFD vs. H-RLPA comparisons. To validate the RNA-seq results, six key genes (Cyp3a11, Cyp2b9, Cyp2b13, Cyp2c40, Cyp26a1 and Ptges) were verified by qRT-PCR in the ND, HFD, H-APAM and H-RLPA groups (Figure 6E), and the expression trends were highly consistent between the two methods, confirming the reliability of the transcriptomic analysis.

### 3.6. Multi-Omics Integration Revealed Associations Linking Gut Microbiota, SCFAs, Hepatic Gene Expression and Metabolic Phenotypes

**Figure 7:**
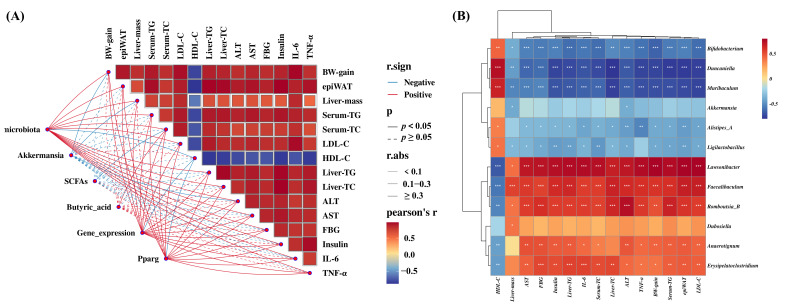
Multi-omics integrative analysis revealing associations among gut microbiota, SCFAs, hepatic gene expression and metabolic phenotypes. (**A**) Mantel test combined with Pearson correlation analysis. The right half-matrix displays Pearson correlation coefficients among 16 metabolic phenotypic indicators. Connecting lines on the left represent Mantel test results between omics datasets (gut microbiota composition, total SCFAs, butyric acid, Akkermansia abundance, hepatic gene expression profiles and Pparg expression) and metabolic parameters. Line color indicates the direction of correlation (red, positive; blue, negative); line thickness represents the absolute Mantel statistic (|r|); and line type denotes statistical significance (solid, *p* < 0.05; dashed, *p* ≥ 0.05). (**B**) Spearman correlation heatmap between differentially abundant genera and metabolic phenotypic parameters. * *p* < 0.05, ** *p* < 0.01, *** *p* < 0.001.

To delineate the interrelationships among changes in gut microbiota, SCFAs, hepatic transcription and host metabolic status, samples from all groups were pooled for Mantel test and Spearman correlation analyses (Figure 7 and Figure S6).

Mantel test (Figure 7A) demonstrated that overall gut microbiota composition, hepatic gene expression profiles and Pparg expression were all significantly associated with the metabolic phenotype matrix (*p* < 0.05), with most associations reaching moderate or higher effect sizes (|r| ≥ 0.3). Total SCFAs and butyric acid were also significantly associated with several metabolic parameters. Akkermansia abundance showed a negative correlation trend with most adverse metabolic indices. Pearson correlation among the 16 metabolic indicators revealed strong positive intercorrelations (r > 0.5) for all indices except HDL-C, which was consistently negatively correlated with the remaining parameters (r < −0.5).

Spearman correlation between differential genera and metabolic phenotypes (Figure 7B) revealed clear bidirectional patterns. Intervention-enriched genera, including Duncaniella, Muribaculum and Bifidobacterium, were highly significantly and negatively correlated with virtually all adverse metabolic indices, while positively correlated with HDL-C (*p* < 0.001). In contrast, HFD-enriched genera including Lawsonibacter, Faecalibaculum and Romboutsia_B showed significant positive correlations with the same adverse parameters (*p* < 0.001). Alistipes_A and Ligilactobacillus were also negatively correlated with most adverse indices (*p* < 0.05–0.01).

The correlation between genera and SCFAs (Figure S6A) further established the functional underpinning of these associations. Intervention-enriched genera were broadly and positively correlated with multiple SCFAs, whereas HFD-enriched genera displayed widespread negative correlations. For instance, Duncaniella and Muribaculum were significantly positively correlated with isobutyric acid, acetic acid and propionic acid (all *p* < 0.01), whereas Faecalibaculum was significantly negatively correlated with the same SCFAs (all *p* < 0.01).

Finally, the correlation between SCFAs and hepatic gene expression (Figure S6B) provided insights into potential gut-liver signaling mechanisms. Pparg was significantly positively correlated with isovaleric acid and isobutyric acid (both *p* < 0.01). Fatty acid oxidation-related genes Ehhadh and Cpt1b were positively correlated with isobutyric acid (*p* < 0.01 and *p* < 0.05, respectively). Hepatic metabolic genes Pck1, Cyp3a11 and Abcb1b were positively correlated with isobutyric acid and propionic acid (*p* < 0.01) but negatively correlated with valeric acid (*p* < 0.01). Inflammation-related genes Ccl2 and Ptges were significantly negatively correlated with isobutyric acid (both *p* < 0.01). These multi-layered correlations collectively revealed an association network linking gut microbiota composition, SCFAs, hepatic gene expression, and metabolic phenotype outcomes.

## 4. Discussion

Obesity is a chronic metabolic disorder characterized by excessive visceral fat accumulation and metabolic dysfunction, which is closely associated with the development of dyslipidemia, insulin resistance, hepatic steatosis and systemic chronic inflammation [27]. Pancreatic lipase inhibition represents a well-established pharmacological strategy for obesity management, as exemplified by the FDA-approved drug Orlistat [4]. Our previous study identified two food-derived peptides, APAM and RLPA, as effective pancreatic lipase inhibitors with distinct IC50 values in vitro, but whether they could exert anti-obesity effects in vivo and through what mechanisms remained unknown. Therefore, in this study, APAM and RLPA were orally co-administered at two dose levels simultaneously with HFD to C57BL/6J mice for 10 weeks, and their effects on obesity-related metabolic parameters, intestinal fat digestion, gut microbiota, fecal SCFAs and hepatic transcriptome were systematically investigated. Compared to the ND group, HFD feeding successfully induced typical obese phenotypes including significant increases in body weight, Lee’s index, visceral fat mass, hepatic steatosis, dyslipidemia, hyperglycemia, hyperinsulinemia and systemic inflammation. Both APAM and RLPA supplementation significantly attenuated HFD-induced obesity and related metabolic disorders in a dose-dependent manner without affecting daily energy intake, suggesting that the anti-obesity effects of the two peptides were not attributable to reduced food consumption but rather to their direct modulation of energy metabolism or fat absorption. Importantly, on most obesity-related phenotypic indices, RLPA groups showed more pronounced improvements than the corresponding APAM groups, with the H-RLPA group exhibiting the most comprehensive anti-obesity effects. The concordance in the overall direction of metabolic improvement between two structurally distinct peptides with different lipase inhibitory potencies strengthened the generalizability and reliability of the findings. Based on the integrated multi-omics analyses, we proposed that these two lipase inhibitory peptides exerted their preventive effects on obesity through a dual-pathway synergistic mechanism encompassing direct inhibition of intestinal fat digestion and indirect regulation of the gut microbiota-SCFAs-liver axis.

The first core mechanism elucidated in this study was the direct inhibition of intestinal fat digestion. Orlistat, the only clinically approved pancreatic lipase inhibitor, reduces dietary fat absorption by approximately 30% through covalent inactivation of gastric and pancreatic lipases, but its clinical utility is limited by gastrointestinal side effects including steatorrhea, fecal urgency and fat-soluble vitamin malabsorption [4,8]. Food-derived lipase inhibitory peptides have therefore emerged as potential safer alternatives. Chen et al. (2024) [9] reported that oyster peptides obtained by simulated gastrointestinal digestion could reduce body weight gain and improve dyslipidemia partly through pancreatic lipase inhibition. Similarly, microencapsulated brewer’s spent grain peptides were shown to preserve their in vivo hypolipidemic activity by enhancing inhibition of both pancreatic lipase and cholesterol esterase [10]. In the present study, both APAM and RLPA significantly reduced the combined pancreatic lipase and cholesterol esterase activities in small intestinal contents and increased fecal TG excretion in a dose-dependent manner, providing direct in vivo evidence for a ‘lipase inhibition-reduced fat absorption-increased fecal fat excretion’ functional link. These in vivo results were consistent with the in vitro IC50 data of the two peptides, demonstrating a clear translation from in vitro enzyme inhibition to in vivo anti-obesity efficacy. However, an intriguing asymmetry was observed, although APAM displayed stronger direct enzyme inhibition than RLPA at each dose level (e.g., H-APAM: 44.3% vs. H-RLPA: 36.8% reduction in enzyme activity), RLPA groups consistently exhibited higher fecal fat excretion (e.g., H-RLPA: 108.4% vs. H-APAM: 73.4% increase over HFD). This dissociation suggested that RLPA might suppress fat absorption through additional mechanisms beyond direct lipase inactivation. Although the exact nature of these supplementary mechanisms was not directly investigated in the present study, possible candidates include interference with bile acid-mediated lipid emulsification, modulation of intestinal lipid transporter expression (such as NPC1L1 and CD36), or alteration of the intestinal physical barrier properties, all of which require dedicated experimental validation in future studies. Notably, RLPA preferentially enriched *Akkermansia muciniphila*, a bacterium reported to strengthen the intestinal mucus layer [28,29], which could theoretically influence the physicochemical conditions of fat absorption, although this interaction was not directly tested in the current study. Additionally, the adaptive upregulation of intestinal enzyme activity in the HFD group (approximately 1.9-fold higher than ND) was consistent with the known compensatory secretory response to increased dietary fat load. These putative supplementary mechanisms warrant direct experimental validation and, if confirmed, could explain why RLPA exhibited more comprehensive anti-obesity phenotypes despite its comparatively weaker direct lipase inhibition.

Accumulating evidence has suggested that alterations in the composition and structure of gut microbiota could affect the host’s energy homeostasis, systemic inflammation, lipid metabolism and insulin sensitivity [30,31]. Multiple studies have demonstrated that food-derived bioactive peptides from diverse sources, including fish collagen [32], walnut [13,14], and quinoa [17], could effectively remodel HFD-disrupted gut microbiota. In the present study, HFD feeding induced a typical dysbiotic signature characterized by reduced α-diversity, an elevated Firmicutes/Bacteroidota (F/B) ratio, enrichment of potentially harmful genera (Desulfovibrio_R, Erysipelatoclostridium, Faecalibaculum) and depletion of beneficial taxa. Both APAM and RLPA supplementation effectively reversed these dysbiotic features, which was consistent with the abovementioned studies. The F/B ratio, widely recognized as a key biomarker for obesity-associated dysbiosis [12,13,17], was significantly normalized by both peptides. It is worth noting that Faecalibaculum, which was dramatically over-represented in the HFD group in our study, was also identified as a characteristic obesity-associated bacterium in previous reports [1]. The excessive enrichment of a single genus reflected the trend toward microbial community homogenization, and peptide supplementation restored the evenness of microbial composition, which was consistent with the improved α-diversity indices. At the genus level, the two peptides enriched distinct sets of beneficial bacteria: APAM preferentially promoted Bifidobacterium and Roseburia, while RLPA characteristically enriched Akkermansia, Lactobacillus and Ligilactobacillus. Akkermansia muciniphila, a next-generation probiotic that colonizes the intestinal mucus layer, has been extensively documented to alleviate obesity, improve insulin sensitivity and strengthen gut barrier function in both animal models and human clinical trials [28,33,34]. Similarly, Li et al. [35] reported that a novel designed peptide D3 could increase the intestinal abundance of A. muciniphila approximately 100-fold through the IFN-γ-Irgm1 axis, further inhibiting fat absorption by downregulating CD36. The enrichment of *Akkermansia* by RLPA may represent a contributing factor to RLPA’s superior systemic metabolic improvements, although a direct causal relationship between *Akkermansia* enrichment and reduced fat absorption was not established in this study. Meanwhile, the reduction in sulfate-reducing bacteria belonging to Desulfobacterota_I was noteworthy, as their metabolic end-product hydrogen sulfide could damage the intestinal barrier and promote inflammation. The decreased abundance of this phylum in peptide-supplemented groups was consistent with the reduced serum levels of IL-6 and TNF-α, suggesting that the suppression of intestinal hydrogen sulfide production might be one of the pathways through which the peptides exerted their anti-inflammatory effects.

These differential microbiota signatures were mirrored in the corresponding SCFA profiles. SCFAs, as one of the major microbial metabolites, are important fuels for intestinal epithelial cells and have been proved to be closely associated with energy metabolism and intestinal homeostasis [15,36]. In this study, APAM groups demonstrated superior restoration of propionic acid levels, which was consistent with the propionate-producing capacity of Roseburia enriched by APAM [37]. In contrast, RLPA groups excelled in butyric acid recovery and effective normalization of branched-chain SCFAs (isobutyric acid and 2-methylbutyric acid). Butyrate is the preferred energy source for colonocytes and plays a pivotal role in maintaining intestinal barrier integrity and suppressing inflammation through GPR109A signaling and histone deacetylase inhibition [38]. The higher butyrate levels in RLPA groups might be mechanistically linked to the enrichment of Akkermansia, although A. muciniphila itself primarily produces acetate and propionate, its mucin-degrading activity could liberate oligosaccharides and acetate that serve as cross-feeding substrates for butyrogenic bacteria [39,40]. Meanwhile, the LEfSe-identified marker genera in RLPA groups, Lactobacillus and Ligilactobacillus, are known lactate producers, and lactate could further serve as a substrate for butyrate-producing bacteria, forming a “lactate to butyrate” metabolic cascade. On the other hand, branched-chain SCFAs are primarily derived from microbial proteolytic fermentation of branched-chain amino acids and are generally considered markers of protein putrefaction associated with gut barrier damage and inflammation [41]. RLPA’s effective normalization of elevated branched-chain SCFAs suggested a suppression of deleterious protein fermentation activity, likely mediated by the reduction in proteolytic genera such as Desulfovibrio_R. Intriguingly, APAM groups did not reduce branched-chain SCFAs and even showed a tendency for further elevation, which might be related to the altered substrate environment in the colon caused by APAM’s stronger lipase inhibition; the increased undigested fat reaching the colon could indirectly change the protein fermentation patterns. These results indirectly reflected that the two peptides shaped distinct microbial metabolic landscapes, and the differential SCFA profiles might contribute to their respective metabolic endpoints through distinct downstream signaling pathways.

The hepatic transcriptome analysis provided a molecular bridge connecting the two upstream pathways to the downstream metabolic phenotype improvements. In this study, the predominantly down-regulatory pattern of DEGs in both peptide groups contrasted with the up-regulatory pattern observed in HFD-responsive DEGs, indicating that the peptides’ primary transcriptomic action was the suppression of HFD-aberrantly activated gene expression programs rather than the induction of novel transcriptional changes. This pattern was consistent with a mechanism of metabolic burden relief; the peptides did not introduce new pharmacological effects but instead restored the hepatic transcriptional landscape toward normality by alleviating the metabolic overload. A total of 134 core co-regulated genes were simultaneously altered by HFD and reversed by both peptides, which likely represented the convergent transcriptional targets of the two peptides through their distinct upstream pathways. This “divergent pathways, convergent endpoints” pattern suggested the existence of a common downstream regulatory node. KEGG pathway analysis identified the PPAR signaling pathway as a core convergence point, significantly enriched in both the ND vs. HFD and HFD vs. H-RLPA comparisons. PPARs, as master transcriptional regulators of fatty acid metabolism, control the expression of genes involved in fatty acid β-oxidation (CPT1A, ACOX1), lipid transport (FABP1, CD36) and lipogenesis (SCD1, FASN), playing pivotal roles in maintaining hepatic lipid homeostasis [42,43]. Similarly, Li et al. (2023) [17] reported that quinoa peptides could alleviate HFD-induced obesity through modulation of the PPAR-α/γ signaling pathway in the liver, and Feng et al. (2026) [2] demonstrated that corn peptides significantly increased PPARγ and PGC-1α expression in adipose tissue while reshaping the gut microbiota. In addition, Chen et al. (2026) [18] showed that sturgeon-derived peptide LLLE reversed permethrin-induced lipid accumulation by modulating cholesterol metabolism and primary bile acid biosynthesis pathways in the liver, further supporting the hepatic transcriptome as a key effector level for anti-obesity peptides. In this study, the multi-omics correlation analysis revealed that Pparg expression was significantly positively correlated with SCFAs (isobutyric acid and isovaleric acid), and fatty acid oxidation genes (Ehhadh, Cpt1b) were positively correlated with propionic acid, while the inflammation-related gene Ptges was negatively correlated with isobutyric acid. These correlations were consistent with the emerging evidence that gut-derived SCFAs, particularly propionate and butyrate, can modulate hepatic PPAR-related gene expression and lipid oxidation programs [16]. Zheng et al. (2023) [16] elegantly demonstrated using GPR41 and GPR43 mice that butyrate directly modulated hepatic lipid metabolism by inhibiting lipogenesis and activating fatty acid oxidation gene expression through the GPR41/43-CaMKII/HDAC1-CREB signaling pathway in primary hepatocytes. Based on these observations, we hypothesized that the hepatic transcriptomic improvements observed in this study were potentially contributed to by two upstream inputs. On one hand, the reduced dietary lipid absorption via lipase inhibition would directly lower the hepatic lipid load from the ‘substrate supply’ perspective; on the other hand, the altered gut microbiota-derived SCFAs, transported to the liver via the portal vein, may modulate the expression of PPAR-related genes from the ‘signal regulation’ perspective. If both pathways operate concurrently, the combined effect of reducing lipid input while potentially promoting lipid oxidation would collectively contribute to the observed reduction in hepatic lipid accumulation. However, it should be noted that this dual-pathway model remains a working hypothesis that requires causal validation. In addition, the bile secretion pathway was uniquely enriched in the HFD vs. H-RLPA comparison, suggesting that bile acid metabolism might also play a role in RLPA-mediated metabolic improvements. This was consistent with the hypothesis proposed earlier that RLPA might interfere with bile acid-mediated lipid emulsification, although direct bile acid metabolomic evidence was needed to confirm this assumption.

An important observation of this study that deserved particular discussion was the relationship between lipase inhibitory potency and gut microbiota improvement. Both APAM and RLPA, despite their markedly different lipase inhibitory activities and consequently different degrees of in vivo fat digestion suppression, exhibited qualitatively similar directions of gut microbiota improvement, including enhanced α-diversity, normalized F/B ratio and enrichment of beneficial genera. Moreover, the magnitude of difference in microbiota improvement between the two peptides was considerably smaller than the corresponding difference in their anti-obesity phenotypes and fecal fat excretion. This observation raised a critical mechanistic question: was the gut microbiota improvement primarily a secondary consequence of lipase inhibition, or did the peptides exert direct prebiotic-like actions on gut bacteria? Several lines of evidence suggested that both direct and indirect mechanisms were likely at play. From the indirect perspective, the overall improvement in host metabolic status (reduced systemic inflammation, improved lipid profiles) resulting from lipase inhibition could create a more favorable intestinal microenvironment for beneficial microbiota. However, a seemingly paradoxical observation argued against lipase inhibition being the sole driver of microbiota improvement: stronger lipase inhibition by APAM diverted more undigested fat to the colon, which would theoretically create an unfavorable environment for many beneficial bacteria that preferentially ferment carbohydrates, yet both peptide groups showed improved microbiota. This paradox implied that the peptides possessed additional microbiota-supporting properties that counteracted the potentially detrimental effects of excess colonic fat. From the direct perspective, small peptides reaching the colon might serve as preferential nitrogen sources for specific beneficial bacteria, selectively promoting their growth in a manner analogous to prebiotics [44]. Furthermore, APAM and RLPA differed in their amino acid sequences, and their hydrolysis products generated by gut microbial enzymes would provide different peptide fragments and amino acids as growth substrates for different bacterial taxa, potentially explaining the distinct sets of beneficial genera enriched by each peptide. It should be acknowledged that the present data could not definitively distinguish between direct and indirect microbiota-modulating effects. Future studies employing in vitro co-culture systems of individual peptides with specific bacterial species, fecal microbiota transplantation (FMT) experiments and germ-free mouse models were needed to dissect the independent contributions of each mechanism and to clarify whether the gut microbiota improvement was an indispensable mediator or merely a bystander of the anti-obesity effects.

However, several limitations of this study should be acknowledged. First, the experimental design did not include normal diet + peptide safety control groups, an Orlistat-treated positive control group, or a scrambled sequence peptide control group. The absence of ND + peptide groups precludes definitive determination of whether the observed effects are exclusively related to HFD-induced metabolic dysfunction, although existing literature suggests that food-derived peptides exert minimal metabolic effects under normal physiological conditions [45]. The lack of an Orlistat control precludes direct benchmarking against the clinical gold standard. The absence of a scrambled sequence control means that generic nitrogen effects cannot be formally excluded, although the sequence-dependent differences in lipase inhibitory potency, microbiota modulation patterns, and SCFA profiles between APAM and RLPA, together with clear dose–response relationships, collectively support sequence-specific bioactivity. Second, although the gastrointestinal stability of both peptides was confirmed using the INFOGEST 2.0 in vitro digestion protocol in a companion study (recovery rates: 89–92% after gastric digestion, 75–85% after complete gastrointestinal digestion), and functional bioavailability was directly supported by significant in vivo intestinal enzyme inhibition (Figure 3A), detailed in vivo pharmacokinetic characterization (absorption, distribution, and elimination) was not performed. Third, the study was conducted exclusively in male mice, limiting generalizability to females that may exhibit different hormonal and metabolic responses. Fourth, the mechanistic insights regarding the gut microbiota-SCFA-hepatic PPAR signaling cascade were derived from correlational multi-omics analyses and require causal validation through approaches such as fecal microbiota transplantation, germ-free mouse models, or SCFA/GPR antagonist experiments. Fifth, although the expression trends of six key DEGs were successfully validated by qRT-PCR (Figure 6E), protein-level confirmation (e.g., Western blot) of transcriptomic findings was not performed in the present study. Future studies should incorporate Western blot or targeted proteomics to verify whether the observed mRNA-level changes in PPAR signaling pathway genes are faithfully reflected at the protein level, given that post-transcriptional and post-translational regulatory mechanisms may modulate the correlation between mRNA abundance and protein expression. Finally, the RNA-seq analysis was performed with a limited number of biological replicates per group. Future studies should incorporate comprehensive control groups, female animals, pharmacokinetic profiling, and causal validation experiments to strengthen and extend the present findings.

In summary, both APAM and RLPA supplementation significantly attenuated HFD-induced body weight gain, visceral fat accumulation, hepatic steatosis, dyslipidemia, hyperglycemia, hyperinsulinemia and systemic inflammation in a dose-dependent manner without affecting daily energy intake. Two peptides markedly reduced intestinal lipase and cholesterol esterase activities and increased fecal fat excretion, supporting the direct inhibition of intestinal fat digestion as a core anti-obesity mechanism. Both peptides effectively reversed HFD-induced gut dysbiosis by normalizing the F/B ratio and enriching distinct sets of beneficial genera, with APAM preferentially promoting Bifidobacterium and Roseburia and RLPA characteristically enriching Akkermansia, Lactobacillus and Ligilactobacillus, accompanied by differential improvements in SCFA profiles. Hepatic transcriptome analysis identified the PPAR signaling pathway as a core convergence point, and multi-omics correlation analysis revealed an association network linking gut microbiota composition, SCFA production, hepatic gene expression, and metabolic phenotypes. On most metabolic parameters, RLPA groups showed more pronounced improvements than APAM groups, likely attributable to RLPA’s superior enrichment of A. muciniphila and additional mechanisms beyond direct lipase inhibition. In conclusion, this study proposed for the first time a dual-pathway working model integrating the direct enzyme inhibitory effects of lipase inhibitory peptides with their potential modulatory effects on the gut microbiota–liver axis in the context of obesity prevention. Our findings indicated the potential of developing food-derived lipase inhibitory peptides as functional food ingredients or dietary supplements for obesity prevention.

## 5. Conclusions

This study demonstrated that two hemp seed protein-derived tetrapeptides, APAM and RLPA, dose-dependently attenuated HFD-induced obesity and associated metabolic complications in male C57BL/6J mice, including visceral fat accumulation, hepatic steatosis, dyslipidemia, hyperglycemia, and systemic inflammation. Both peptides reduced total intestinal lipid digestive enzyme activity and increased fecal lipid excretion, supporting direct inhibition of intestinal fat digestion as a primary anti-obesity mechanism. Concurrently, both peptides partially restored HFD-disrupted gut microbiota composition by normalizing the *Firmicutes/Bacteroidota* ratio and selectively enriching distinct beneficial genera (*Bifidobacterium* and Roseburia for APAM; Akkermansia, Lactobacillus, and *Ligilactobacillus* for RLPA), accompanied by differential improvements in fecal SCFA profiles. Hepatic transcriptome analysis identified the PPAR signaling pathway as a shared regulatory node, and multi-omics correlation analysis revealed an association network linking gut microbiota composition, SCFA production, hepatic gene expression, and metabolic phenotypes. These findings collectively support a dual-pathway working model in which food-derived lipase inhibitory peptides may prevent HFD-induced obesity through direct intestinal enzyme inhibition combined with potential modulation of the gut microbiota-SCFA-hepatic PPAR axis, although causal validation of the latter pathway remains to be established. This investigation highlights the potential of hemp seed protein-derived peptides as functional food ingredients for dietary obesity prevention.

Several limitations should be acknowledged. The experimental design lacked ND + peptide safety controls, an Orlistat-treated positive control, and scrambled sequence peptide controls; only male mice were studied; detailed peptide pharmacokinetics were not characterized despite confirmed in vitro gastrointestinal stability; transcriptomic findings were validated at the mRNA level by qRT-PCR but not at the protein level; and the proposed gut microbiota-SCFA-hepatic PPAR mechanistic cascade requires causal validation through approaches such as fecal microbiota transplantation or germ-free mouse models. Future studies incorporating these elements are needed to establish the full translational potential of these findings.

## Data Availability

Raw 16S rRNA gene amplicon sequencing data have been deposited in the NCBI Sequence Read Archive (SRA) under BioProject accession PRJNA1470247. Hepatic transcriptome sequencing data have been deposited in the NCBI Sequence Read Archive (SRA) under BioProject accession PRJNA1474678. The other data supporting the conclusions of this article will be made available by the authors on request.

## Supplementary Materials

The following supporting information can be downloaded at: https://www.mdpi.com/article/10.3390/foods15112040/s1, Table S1: Compositions of experimental diets; Table S2: Primer sequences for real-time *qPCR*; Figure S1: High performance liquid chromatography (A) and mass spectrometry (B) plots of APAM. High performance liquid chromatography (C) and mass spectrometry (D) plots of RLPA; Figure S2: Effects of APAM and RLPA supplementation on HFD-induced obesity in mice. (A) epiWAT mass. (B) H&E stained images of epiWAT sections. Data are expressed as mean ± SD (*n* = 8). * *p* < 0.05, ** *p* < 0.01, *** *p* < 0.001, **** *p* < 0.0001; ns, not significant; Figure S3: Supplementary analysis of gut microbial diversity and community structure. (A) Alpha diversity indices (Simpson and Observed species). (B) Non-metric multidimensional scaling (NMDS) analysis based on Bray-Curtis dissimilarity at the genus level. (C) Venn diagram showing shared and unique amplicon sequence variants (ASVs) among the six groups. (D) Hierarchical clustering analysis based on Bray–Curtis distance. Data are expressed as mean ± SD; Figure S4: Supplementary analysis of gut microbial taxonomic composition and predicted functional profiles. (A) Number of observed species across groups. (B) Relative abundance of gut microbiota at the species level. (C) LEfSe analysis (LDA threshold = 2.63) identifying differentially enriched taxa among the six groups. (D) Predicted functional profiles of gut microbiota based on KEGG pathway analysis; Figure S5: Supplementary hepatic transcriptome analysis. (A) Principal component analysis (PCA) of hepatic transcriptome profiles across all groups. (B) Heatmap with hierarchical clustering of DEGs across all groups. (C) Gene Ontology (GO) enrichment analysis of DEGs in ND vs. HFD (top), HFD vs. H-APAM (middle) and HFD vs. H-RLPA (bottom) comparisons. Figure S6: Multi-omics integrative analysis revealing associations among gut microbiota, SCFAs, hepatic gene expression and metabolic phenotypes.
