# Supplementary material for: Hemp Seed Protein-Derived Lipase Inhibitory Peptides Attenuate High-Fat Diet-Induced Obesity: Evidence from Intestinal Fat Digestion and Gut–Liver Axis Regulation
Source: Foods. 2026 Jun 5;15(11):2040. doi: 10.3390/foods15112040 (PMC13257395; doi:10.3390/foods15112040)
Supplement: Supplementary file 1 [file foods-15-02040-s001.zip › foods-4313580-supplementary.pdf]

Supplementary Materials

Table S1 Compositions of experimental diets

Ingredients	Normal diet XTCON50J (g)	High fat diet XTHF60 (g)
Casein	200	200
L-Cystine	3.00	3.00
Corn Starch	506.20	0
Maltodextrin	125.00	125
Sucrose	72.8	72.8
Cellulose	50	50
Soybean Oil	25	25
Lard	20	245
Mineral Mix S10026B	50	50
Vitamin Mix V10001C	1	1
Choline Bitrartrate	2	2
FD&C Yellow Dye #5	0.04	0.00
FD&C Blue Dye #1	0.01	0.05

Values represent grams of each ingredient per formulation batch (total batch weight: 1055.05 g for the normal diet, 773.85 g for the high-fat diet).

Table S2 Primer sequences for real-time *qPCR*

Genes	Forward sequences（5′-3′）	Reverse sequences（5′-3′）
β-actin	GTGACGTTGACATCCGTAAAGA	GTAACAGTCCGCCTAGAAGCAC
Cyp2b9	GGGAGTCCTGCTCATGCTCAAGT	CACCTGATCAATCTCCTTTTGGA
Cyp2b13	AGCTCTCCATGACCCACAGT	GGGAGGATGGGACGTGAAGAAA
Ptges	GGATGCGCTGAAACGTGGA	CAGGAATGAGTACACGAAGCC
Cyp3a11	ACAAACAAGCAGGGATGGAC	GGTAGAGGAGCACCAAGCTG
Cyp26a1	CTCGCACAAGCAGCGAAAG	GATCACGGGCACGTAGCACT
Cyp2c40	TGTATGCAGGACAGGAACCAC	TGCTGAGAAAGGCACGAAGT

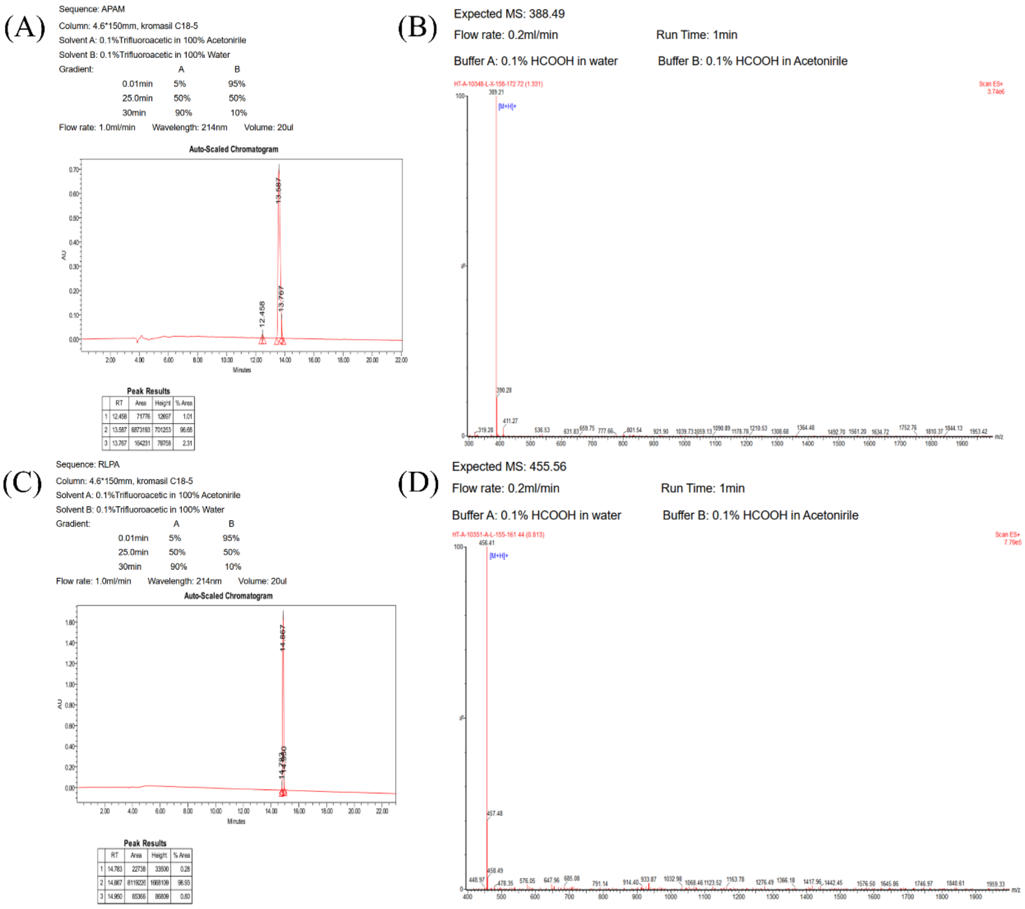

Fig. S1 High performance liquid chromatography (A) and mass spectrometry (B) plots of APAM. High performance liquid chromatography (C) and mass spectrometry (D) plots of RLPA.

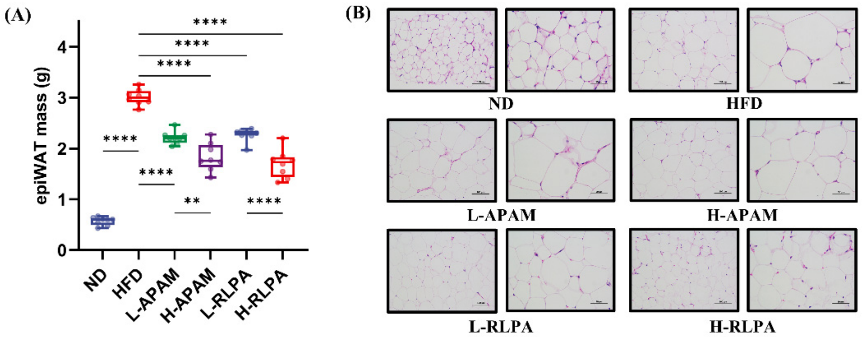

Fig. S2. Effects of APAM and RLPA supplementation on HFD-induced obesity in mice. (A) epiWAT mass. (B) H&E stained images of epiWAT

sections. Data are expressed as mean ± SD (n = 8). **p < 0.01, ****p < 0.0001.

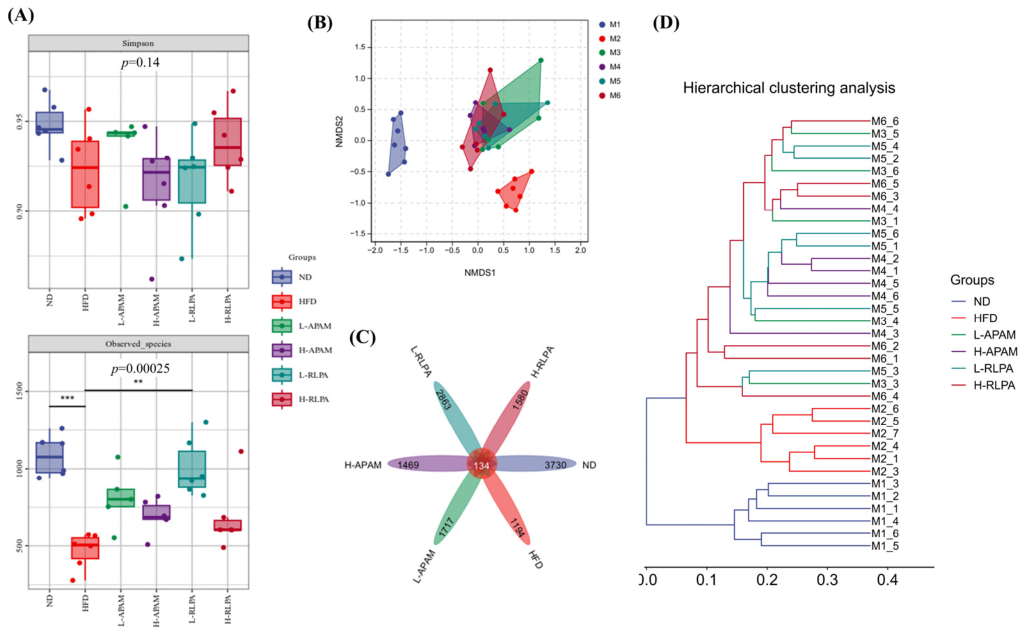

Fig. S3. Supplementary analysis of gut microbial diversity and community structure. (A) Alpha diversity indices (Simpson and Observed species). (B) Non-metric multidimensional scaling (NMDS) analysis based on Bray-Curtis dissimilarity at the genus level. (C) Venn diagram showing shared and unique amplicon sequence variants (ASVs) among the six groups. (D) Hierarchical clustering analysis based on Bray–Curtis distance. Data are expressed as mean ± SD.

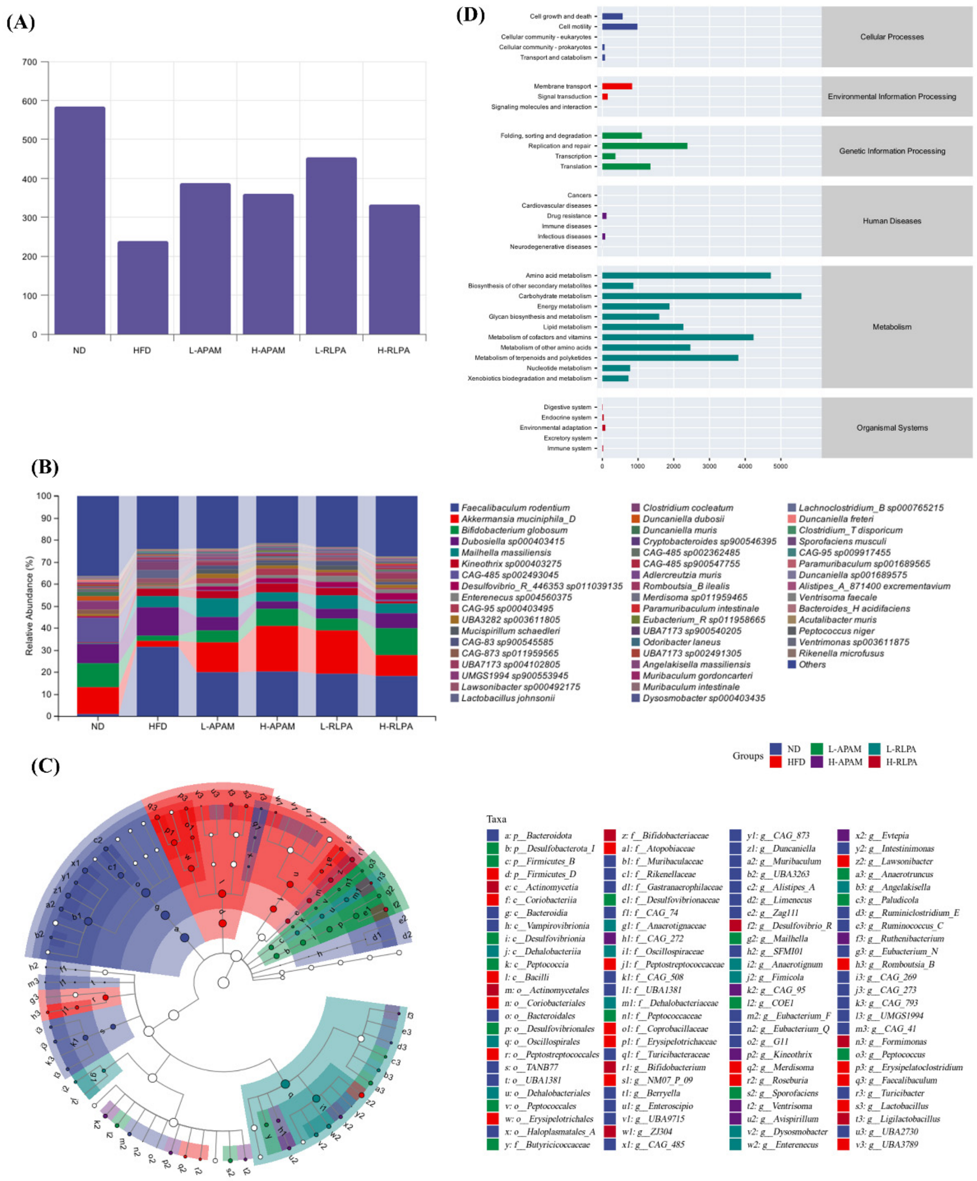

Fig. S4. Supplementary analysis of gut microbial taxonomic composition and predicted functional profiles. (A) Number of observed species across groups. (B) Relative abundance of gut microbiota at the species level. (C) LEfSe analysis (LDA threshold = 2.63) identifying differentially enriched taxa among the six groups. (D) Predicted functional profiles of gut microbiota based on KEGG pathway analysis.

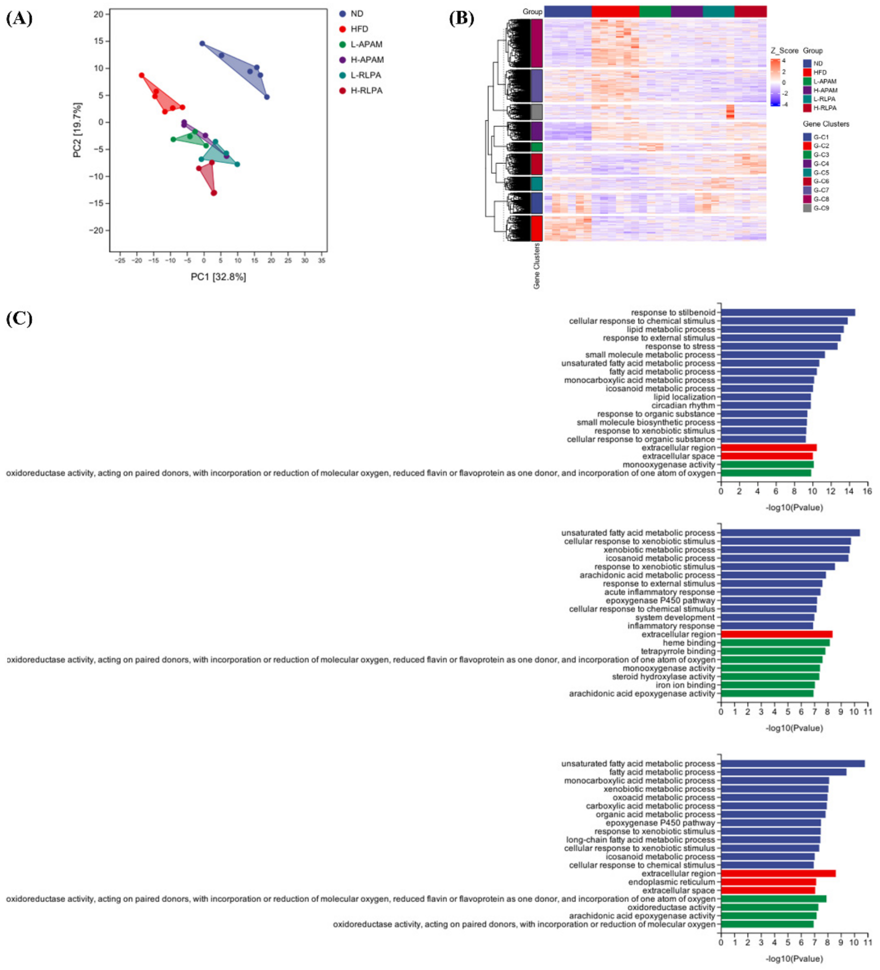

Fig. S5 Supplementary hepatic transcriptome analysis. (A) Principal component analysis (PCA) of hepatic transcriptome profiles across all groups. (B) Heatmap with hierarchical clustering of DEGs across all groups. (C) Gene Ontology (GO) enrichment analysis of DEGs in ND vs. HFD (top), HFD vs. H-APAM (middle) and HFD vs. H-RLPA (bottom) comparisons.

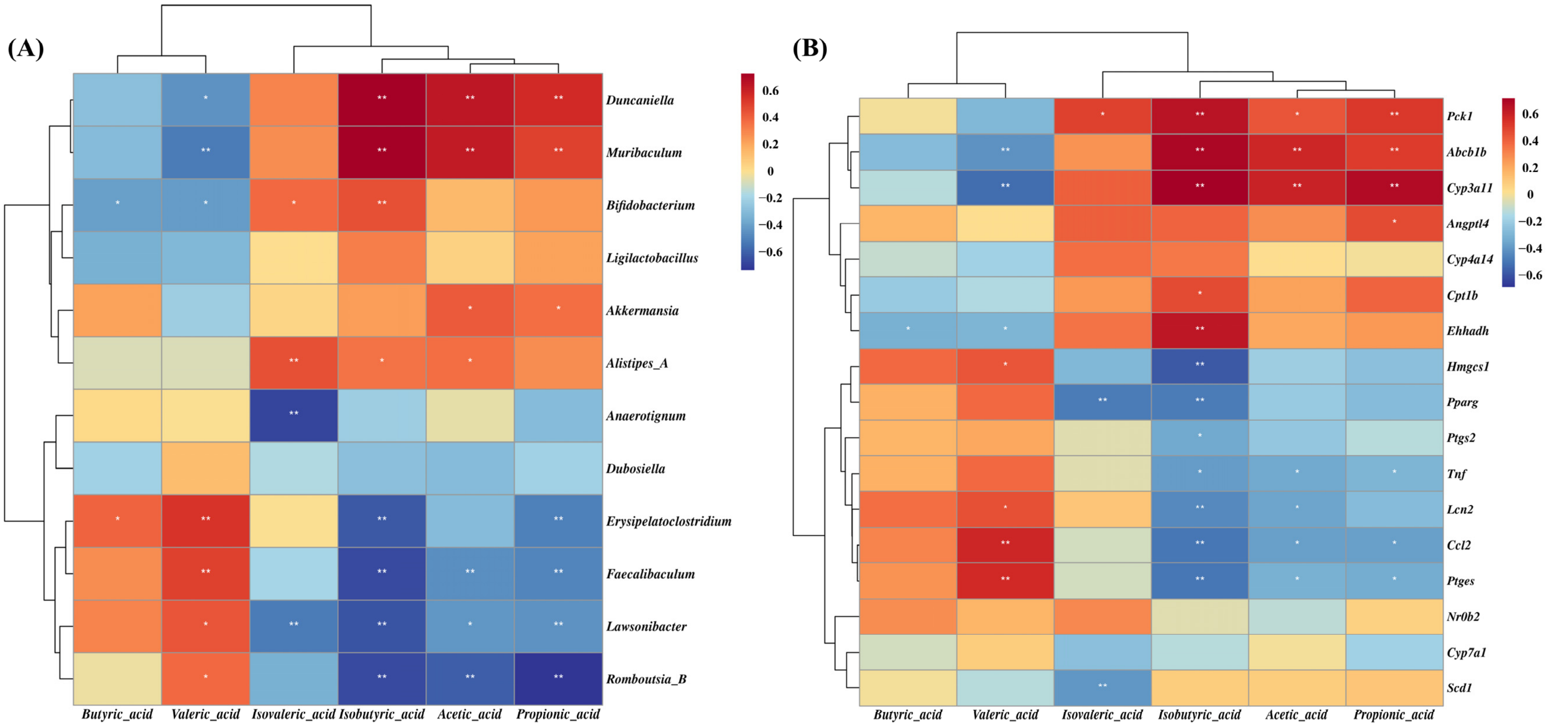

Fig. S6. Multi-omics integrative analysis revealing associations among gut microbiota, SCFAs, hepatic gene expression and metabolic phenotypes. (A) Spearman correlation heatmap between differentially abundant genera and fecal SCFAs. (B) Spearman correlation heatmap between fecal SCFAs and hepatic DEGs from key KEGG pathways. *$p < 0.05$, **$p < 0.01$.
